# *Curcuma phaeocaulis* Inhibits NLRP3 Inflammasome in Macrophages and Ameliorates Nanoparticle-Induced Airway Inflammation in Mice

**DOI:** 10.3390/molecules27072101

**Published:** 2022-03-24

**Authors:** Yeon-Ju Nam, Jiwon Choi, Jong Suk Lee, Changon Seo, Gyeongbeen Lee, Youngsu Lee, Jin Kyu Kim, Pansoo Kim, Jeong Ju Lim, Hyeon-Son Choi, Yongmun Choi

**Affiliations:** 1 Biocenter, Gyeonggido Business and Science Accelerator, Suwon 16229, Korea; nyj@gbsa.or.kr (Y.-J.N.); leejs@gbsa.or.kr (J.S.L.); changon2@gbsa.or.kr (C.S.); bin@gbsa.or.kr (G.L.); gju6020@naver.com (Y.L.); jinkyu90@gbsa.or.kr (J.K.K.); pan@gbsa.or.kr (P.K.); 2 College of Pharmacy, Dongduk Women’s University, Seoul 02748, Korea; edccjw@gmail.com; 3 Interdisciplinary Program in Precision Public Health, Korea University, Seoul 02841, Korea; hdgmp42jj@naver.com; 4 Department of Food Nutrition, Sangmyung University, Seoul 03016, Korea; hsc1970@smu.ac.kr

**Keywords:** NLRP3, inflammasome, *Curcuma phaeocaulis* Valeton, interleukin-1β, curcumin

## Abstract

The activation of NLRP3 results in the assembly of inflammasome that regulates caspase-1 activation and the subsequent secretion of bioactive interleukin (IL)-1β. Excessive activation of the NLRP3 inflammasome is mechanistically linked to diverse pathophysiological conditions, including airway inflammation. Here, we discovered that *Curcuma phaeocaulis* can suppress caspase-1 activation and processing of pro-IL-1β into mature cytokine in macrophages stimulated with NLRP3 inflammasome activators, such as SiO_2_ or TiO_2_ nanoparticles. Furthermore, in the bronchoalveolar lavage fluids of animals administered the nanoparticles, the in vitro effects of *C. phaeocaulis* translated into a decrease in IL-1β levels and cell infiltration. Demethoxycurcumin (DMC) and curcumin were found to be responsible for the inflammasome inhibitory activity of *C. phaeocaulis*. Interestingly, in contrast to the previously reported higher antioxidant- and NFκB-inhibitory activities of curcumin, DMC exhibited approximately two-fold stronger potency than curcumin against nanoparticle induced activation of NLRP3 inflammasome. In the light of these results, both compounds seem to act independently of their antioxidant- and NFκB-inhibitory properties. Although how *C. phaeocaulis* inhibits nanoparticle-activated NLRP3 inflammasome remains to be elucidated, our results provide a basis for further research on *C. phaeocaulis* extract as an anti-inflammatory agent for the treatment of disorders associated with excessive activation of NLRP3 inflammasome.

## 1. Introduction

Inflammatory responses are initiated by the interaction of pathogenic molecules or danger signals with pattern recognition receptors (PRRs) in innate immune cells. Nucleotide-binding oligomerization domain-like receptors (NLRs) are among the PRRs that sense various ligands from microbial pathogens, host cells, and environmental sources, such as asbestos, silica particles, etc. [1,2]. The human NLR gene family is composed of 22 members [3], and of particular interest regarding certain members of the NLRs is the ability to form intracellular protein platforms, named inflammasome, after sensing the intracellular changes caused by danger signals [4]. NLRP3 is a well-characterized NLR, and it undergoes self-oligomerization upon activation, recruiting the adaptor protein ASC (apoptosis spec-like protein containing a caspase recruitment domain) and pro-caspase-1. The resulting NLRP3 inflammasome induces caspase-1 cleavage and processing of pro-interleukin (IL)-1β into the mature IL-1β [5].

Some dangerous signals have been reported to activate NLRP3 inflammasome in various tissues and organs. For instance, in the respiratory tract tissue, SiO_2_ and TiO_2_ nanoparticles have been reported to trigger inflammatory responses involving IL-1β secretion in an NLRP3 inflammasome-dependent manner [6,7,8,9,10]. Of particular interest is the observation that both nanoparticles were able to synergistically induce the activation of macrophages at concentrations which neither of the nanoparticles alone was capable of activating inflammasome in animals [7]. These observations can be exploited to evaluate the efficacy of inflammasome inhibitors because relatively low doses of the nanoparticles may reduce toxicity without compromising the ability of the nanoparticles to activate inflammasome.

Excessive activation of NLRP3 inflammasome has been implicated in the development and aggravation of inflammatory diseases including airway inflammation. Therefore, it is considered as a promising therapeutic target in respiratory diseases associated with inflammation. In fact, several natural products and chemical compounds have been identified to inhibit the formation of NLRP3 inflammasome in vitro and in animal models of respiratory diseases [11,12]. However, the clinical applications of the reported inhibitors remain limited due to undefined pharmacology or poor pharmacokinetic properties of the inhibitors.

In this study, therefore, in an effort to discover new pharmacological modalities of NLRP3 inhibition, we performed a screening campaign of extracts derived from herbal medicinal plants in the Korean Herbal Pharmacopoeia (KHP). The screening campaign and in vivo functional analyses in an animal model of nanoparticle-induced airway inflammation resulted in the identification of *Curcuma phaeocaulis* Valeton (Zingiberaceae family). These results provide a basis for further characterization of *C. phaeocaulis* extract as an anti-inflammatory agent for the treatment of the disorders caused by the excessive activation of NLRP3 inflammasome.

## 2. Results

### 2.1. In Vitro Identification of the Extract of C. phaeocaulis as an Inhibitor of NLRP3 Inflammasome

**Figure 1:**
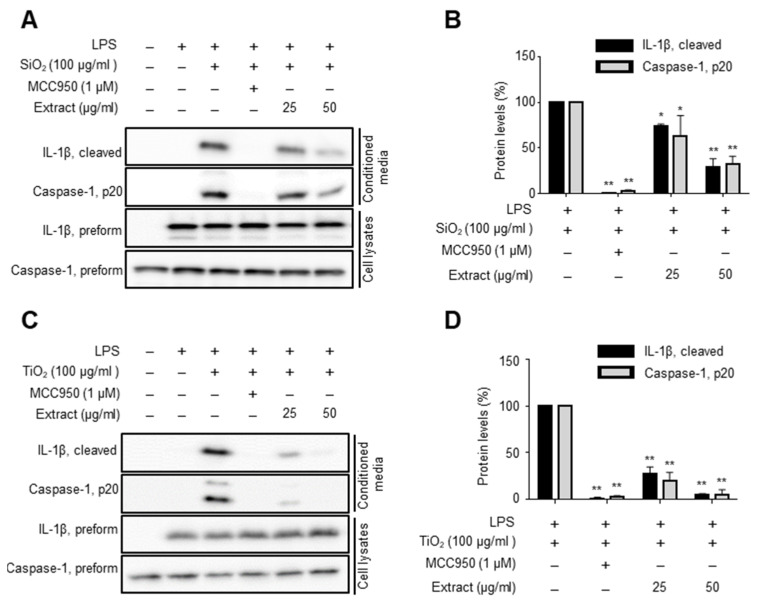
Identification of *C. phaeocaulis* extract as an inhibitor of NLRP3 inflammasome in vitro. Bone marrow-derived mononuclear cells were differentiated into macrophages. The cells were primed with lipopolysaccharide for 2 h, and then they were pretreated with either DMSO, MCC950, or extract for 1 h before inflammasome activation by either SiO_2_ (**A**,**B**), or TiO_2_ (**C**,**D**) nanoparticles. The conditioned media and total lysates were used for Western blot analysis (left). Data from three independent replicates are shown for cleaved IL-1β and caspase-1 (p20) as bar graphs. (right). Data are expressed as mean ± standard deviation (SD), and different symbols indicate differences at * *p* < 0.05, ** *p* < 0.001 vs. LPS + SiO_2_, or LPS + TiO_2_ group.

In order to screen for the inhibitors of NLRP3 inflammasome, we used macrophages that were differentiated from human bone marrow derived-primary mononuclear cells in vitro. The cells were primed with lipopolysaccharide and treated with SiO_2_, and then the secreted IL-1β in cultured media was analyzed using ELISA as a readout of the inflammasome activation. For the screening campaign, a library of extracts derived from herbal medicinal plants was tested in duplicates at a concentration of 100 μg/mL for their ability to attenuate SiO_2_-induced secretion of IL-1β active form (Figure S1, Supplementary Material). MCC950 was used as a reference compound [13]. Eventually, we identified, among others, *C. phaeocaulis* extract as a promising candidate. Western blot analysis of conditioned media derived from the macrophages treated with either DMSO or *C. phaeocaulis* extract confirmed that the levels of the secreted active form of IL-1β and caspase-1 activity were reduced in *C. phaeocaulis*-treated cells as compared with those in the DMSO-treated cells (Figure 1A). At a concentration of 50 μg/mL, *C. phaeocaulis* extract reduced the levels of secreted IL-1β and caspase-1 by 70.7% and 67.4%, respectively, whereas the extract had little effect on the expression of IL-1β and caspase-1 preforms (Figure 1B).

Subsequently, we tested whether *C. phaeocaulis* extract inhibited TiO_2_-activated NLRP3 inflammasome in the macrophages. As shown in Figure 1C, the levels of TiO_2_-induced cleaved forms of IL-1β and caspase-1 were significantly attenuated by *C. phaeocaulis* extract as compared with those in TiO_2_-only treated cells. Specifically, at a concentration of 50 μg/mL, *C. phaeocaulis* extract reduced the levels of secreted IL-1β and caspase-1 by 95.5% and 94.8%, respectively (Figure 1D). However, as expected, the protein levels of IL-1β and caspase-1 preforms in the cellular extracts were not affected by *C. phaeocaulis*. These results indicate that *C. phaeocaulis* extract inhibited SiO_2_- and TiO_2_-induced activation of NLRP3 inflammasome in macrophages.

### 2.2. Identification of Active Compounds in C. phaeocaulis Extract

**Figure 2:**
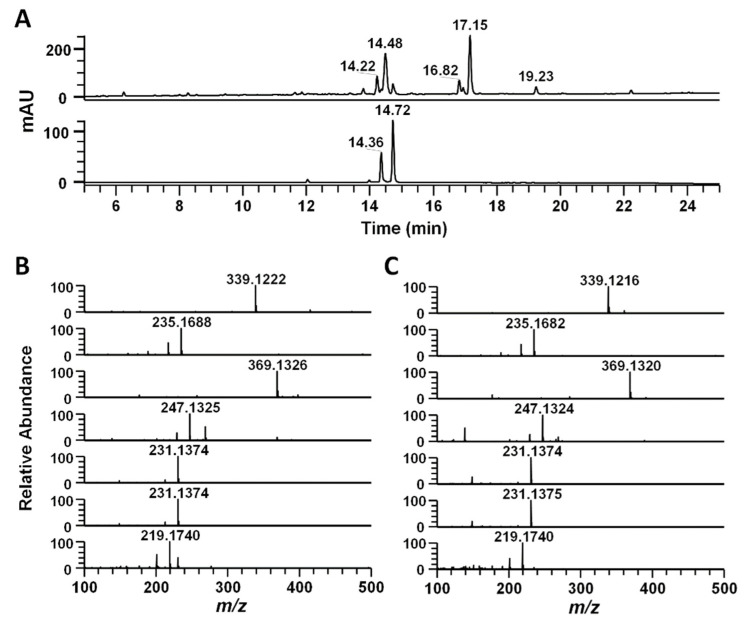
The UHPLC-HRMS analyses of *C. phaeocaulis* extract. (**A**) UHPLC-PDA chromatogram of the extract. The high-resolution mass spectra of seven main peaks (**B**) and the standard compounds (**C**) are shown.

In order to identify the major bioactive ingredients that contribute to the inflammasome-inhibitory activity of *C. phaeocaulis*, we employed a dereplication strategy to determine the compounds that are present in the extract. The dereplication strategy involves liquid chromatography-mass spectrometry, high-resolution mass spectral data, and MS/MS spectral data, which were used to search online natural product databases and our in-house MS/MS spectral library. The efforts led to the identification of seven compounds, including demethoxycurcumin, curcumenol, curcumin, zederone, curzerenone, furanodienone, and germacrone (Figure 2A and Table 1). By comparison with mass data of commercially obtained standards, their identities were confirmed (Figure 2B,C).

**Figure 3:**
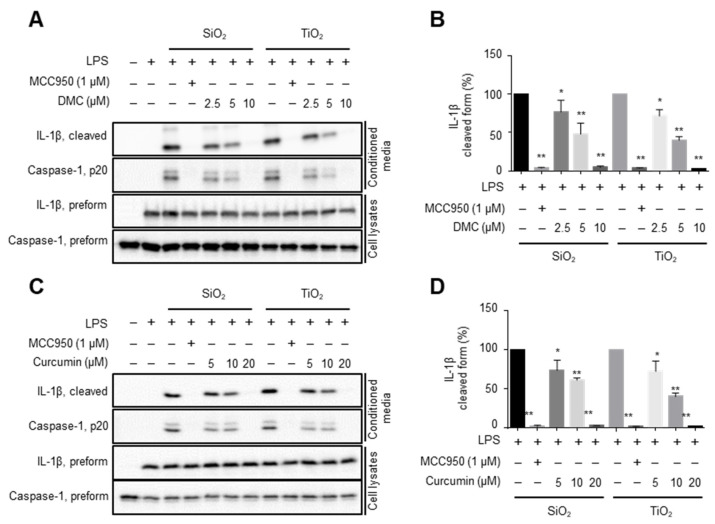
DMC and curcumin are active ingredients in *C. phaeocaulis* extract. Bone marrow-derived mononuclear cells were differentiated into macrophages. The cells were primed with lipopolysaccharide and treated with either SiO_2_ or TiO_2_ to activate inflammasome. The effects of DMC on the activation of inflammasome was determined by analyzing the levels of the secreted IL-1β and caspase-1 (p20) active forms in cultured media by Western blot (**A**). Data from three independent replicates are shown for cleaved IL-1β as bar graphs (**B**). The effects of curcumin on the activation of inflammasome was determined by analyzing the levels of the secreted IL-1β and caspase-1 (p20) active forms in cultured media by Western blot (**C**). Data from three independent replicates are shown for cleaved IL-1β as bar graphs (**D**). Data are expressed as mean ± SD, and different symbols indicate differences at * *p* < 0.05, ** *p* < 0.001 vs. LPS + SiO_2_ or LPS + TiO_2_ group.

As a follow-up, the identified compounds were tested for their ability to inhibit nanoparticle-activated NLRP3 inflammasome in the macrophages by ELISA. At a concentration of 25 µM, only demethoxycurcumin (DMC) and curcumin were able to almost completely attenuate the levels of the cleaved forms of IL-1β in conditioned media derived from SiO_2_-treated macrophages; however, other compounds exhibited little effects on the levels of the cleaved forms of IL-1β (Supplementary Figure S2). Titration of DMC and curcumin revealed that DMC inhibited the nanoparticle-induced IL-1β and caspase-1 cleavage with an IC_50_ of less than 5 μM (Figure 3A,B). In fact, at a concentration of 10 μM, DMC almost completely inhibited the nanoparticle-induced inflammasome activity, while curcumin, at a concentration of 10 μM, inhibited SiO_2_-induced IL-1β cleavage by approximately 40% (Figure 3C,D).

### 2.3. Molecular Docking Studies for DMC and Curcumin

In order to gain insights into how DMC and curcumin inhibited NLRP3 inflammasome with different profiles of efficacy, we performed molecular docking studies to assess the possibility of each of NLRP3 inflammasome components as a molecular target of the inhibitors. The crystal structure of each component, including the pyrin domain (PYD) of ASC (2KN6) and NLRP3 NACHT (7ALV), was retrieved from RCSB Protein Data Bank (PDB). Then, the SiteMap module of the Schrödinger software package was used to explore potential ligand binding sites in the protein structures. Following molecular docking of DMC and curcumin to the identified ligand binding sites, we used the Glide docking scores as a measure of binding affinity and selectivity of the compounds. The results indicated that the best docking scores of DMC and curcumin at the binding site of the PYD of ASC protein were −5.037 kcal/mol and −3.996 kcal/mol, respectively (Supplementary Figure S3), whereas the scores at the NLRP3 NACHT domain were −6.44 kcal/mol and −6.02 kcal/mol, respectively. Albeit, additional supporting data are absolutely required as NLRP3 NACHT domain is more likely to be a target of the inhibitors than PYD of ASC.

**Figure 4:**
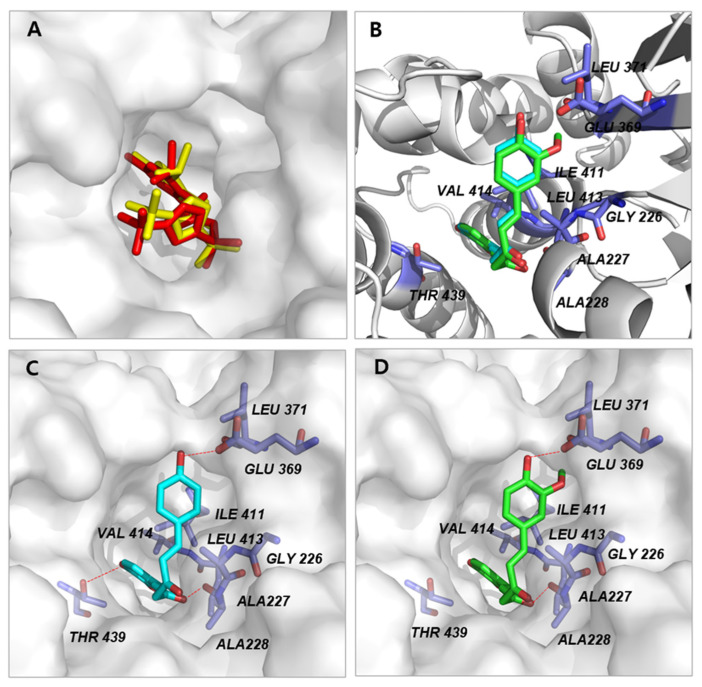
Molecular docking of DMC and curcumin to NLRP3 protein. (**A**) The co-crystal ligand, NP3-146 can be well re-docked into its own binding pocket with RMSD = 0.353 Å. The co-crystal NP3-146 in NLRP3 NACHT domain are indicated by the red stick. (**B**) Superimposition of predicted conformations for DMC (cyan) and curcumin (green) in the NLRP3 NACHT domain structure. (**C**) Detailed interactions between DMC and NLRP3 protein are shown as a stick model, and hydrogen bonds are indicated with red dashed lines. There are three possible hydrogen bond between DMC and NLRP3 protein, i.e., C12-OH with Glu369, C13-OH with Thr439, and the C15-OH with Ala228. (**D**) Detailed interactions between curcumin and NLRP3 protein are shown as a stick model, and H-bonds are indicated with red dashed lines. Figures were drawn by using PyMol (Delano Scientific LLC, San Carlos, CA, USA).

The ligand-binding site in the NLRP3 NACHT domain that we used in the current study was also reported to be a binding pocket for an NLRP3 inhibitor NP3-146 [14]. Furthermore, the molecular docking analysis of NP3-146 against the ligand-binding site showed excellent superimposition of the predicted pose with the experimental crystal ligand (RMSD = 0.0367 Å) (Figure 4A). This indicates that our docking algorithm is well-suited for the molecular modeling. In the predicted binding pose, the overall backbone conformation for DMC and curcumin were similar (Figure 4B); the carboxyl group of E369 and A228 in NLRP3 NACHT domain form hydrogen bonds with both DMC and curcumin (Figure 4C,D). However, while the side chain of T439 forms a hydrogen bond with 2-methoxyphenol group of DMC, curcumin failed to make interaction with T439. These observations could explain the observed different efficacy profiles of DMC and curcumin against NLRP3 inflammasome in vitro. It remains to provide additional evidences including in vitro experimental data to support the proposed docking models.

### 2.4. Effects of C. phaeocaulis Extract on the Nanoparticle-Induced Airway Inflammation in Mice

**Figure 5:**
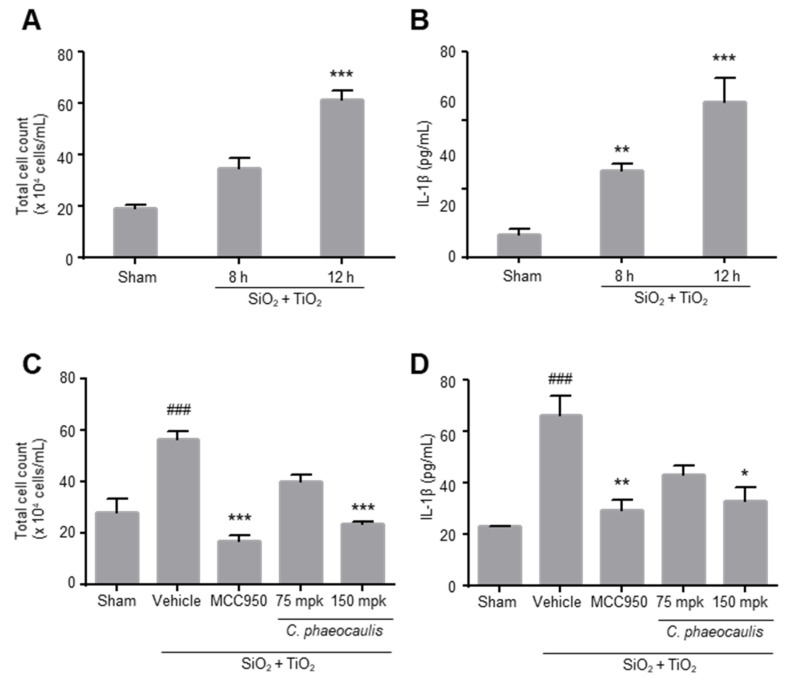
The effects of *C. phaeocaulis* extract on the nanoparticle-induced airway inflammation in mice. SiO_2_ (20 mg/kg) and TiO_2_ (20 mg/kg) were administered by intratracheal instillation following anesthesia with pentobarbital. At 8 or 12 h after the nanoparticle instillation, BALFs were analyzed for total cell counts (**A**) and IL-1β (**B**). Data are expressed as mean ± standard error (SE), and different symbols indicate differences at ** *p* < 0.01, *** *p* < 0.001 vs. sham group. (**C**,**D**) *C. phaeocaulis* extract was orally administered 1 h before and 4 h after intratracheal instillation of nanoparticles. At 12 h after the nanoparticle instillation, BALFs were analyzed for total cell counts and IL-1β, respectively. MCC950 was used as a reference compound inhibiting NLRP3 inflammasome. Data are expressed as mean ± SE, and different symbols indicate differences at ^###^ *p* < 0.001 vs. sham group; * *p* < 0.05, ** *p* < 0.01, *** *p* < 0.001 vs. SiO_2_ + TiO_2_ + vehicle administered group.

Both SiO_2_ and TiO_2_ nanoparticles have been reported to trigger IL-1β secretion in the lung and provoke airway inflammation in animals [15]. Meanwhile, the neutralization of IL-1β has been shown to attenuate silica-induced lung inflammation [8]. Therefore, we probed whether *C. phaeocaulis* extract can produce the desired biological outcome in an animal model of airway inflammation caused by these nanoparticles. In order to establish an animal model, both SiO_2_ and TiO_2_ nanoparticles were administered by intratracheal instillation. Subsequently, the total cell number and the levels of IL-1β in bronchoalveolar lavage fluids (BALFs) were analyzed. As shown in Figure 5A,B, following 12 h after the instillation of SiO_2_ and TiO_2_, both total cell number and IL-1β levels in BALFs were significantly increased compared with those in sham control animals.

In this established animal model, *C. phaeocaulis* extract was orally administered 1 h before and 4 h after intratracheal instillation of SiO_2_ and TiO_2_ nanoparticles. MCC950 (10 mg/kg, i.p. injection) was used as a reference compound. Analysis of BALFs at 12 h after nanoparticle instillation showed that the nanoparticle-induced increases in inflammatory cell counts in BAL fluids were markedly suppressed by MCC950 and *C. phaeocaulis* extract. At a dose of 150 mg/kg, the extract reduced the total inflammatory cell counts to the levels observed in control animals (sham control: 27.81 ± 5.72 cells/mL, nanoparticle instillation: 56.38 ± 3.27 cells/mL, MCC950: 17.06 ± 2.24 cells/mL, and *C. phaeocaulis*: 23.38 ± 0.84 cells/mL) (Figure 5C). In addition, MCC950 and *C. phaeocaulis* extract (150 mg/kg) significantly decreased the nanoparticle-induced increases in IL-1β in BALFs (nanoparticle instillation: 66.33 ± 7.49 pg/mL, MCC950: 29.56 ± 3.86 pg/mL, and *C. phaeocaulis*: 32.95 ± 5.47 pg/mL) (Figure 5D).

## 3. Discussion

SiO_2_ and TiO_2_ nanoparticles are environmentally hazardous substances that have been reported to activate the NLRP3 inflammasome in vitro and in vivo [16]. Here, we identified *Curcuma phaeocaulis* as an inhibitor of SiO_2_ and TiO_2_ nanoparticle-activated NLRP3 inflammasome. Although how *C. phaeocaulis* extract inhibits nanoparticle-activated NLRP3 inflammasome has not been elucidated, *C. phaeocaulis* was demonstrated to suppress the nanoparticle-induced increases in the active proteins of IL-1β and caspase-1 in macrophages. In addition, the in vitro effects of *C. phaeocaulis* translate into a decrease in IL-1β levels and inflammatory cell infiltration in an animal model of airway inflammation caused by nanoparticles. Considering the fact that NLRP3 inflammasome is mechanistically linked to diverse pathophysiological conditions, including insulin resistance and neuroinflammation [17,18], it will be scientifically worthwhile to evaluate *C. phaeocaulis* extract in other animal models of diseases associated with excessive activation of NLRP3 inflammasome.

DMC and curcumin turned out to contribute to the ability of the extract of *C. phaeocaulis* to inhibit NLRP3 inflammasome activated by SiO_2_ and TiO_2_ nanoparticles (Figure 3). Moreover, both compounds (DMC and curcumin) were shown to inhibit NLRP3 inflammasome activated by nigericin, imiquimod, and monosodium urate crystals in murine macrophages and human monocytes. However, neither the NLRC4 inflammasome nor AIM2 inflammasome were inhibited by curcumin [19]. It is unlikely that both inhibitors prevent lysosomal damage or mitochondrial ROS generation, because only SiO_2_, but not TiO_2_, induces lysosomal damage in macrophages [7]. In addition, blocking of the generation of ROS by an antioxidant (butylated hydroxyanisole) only slightly inhibited nanoparticle induced IL-1β secretion in macrophages [7]. Instead, most NLRP3 inflammasome activators appear to share K^+^ efflux as the common intermediate event leading to NLRP3 inflammasome assembly [20]. Therefore, it is reasonable to speculate that both DMC and curcumin may regulate downstream cellular events induced by K^+^ efflux or modulate K^+^ efflux induced by NLRP3 inflammasome activators.

Curcumin differs from DMC by the substitution pattern of ortho-methoxy group on the aromatic ring: curcumin contains two phenyl methoxy groups, while DMC has one. In the present study, DMC was slightly (~2 fold) more potent than curcumin in inhibiting the cleavage of IL-1β and caspase-1 induced by nanoparticles in macrophages. Conversely, other research groups previously reported that curcumin exhibited stronger antioxidant activity than DMC [21]. In addition, TNFα-induced NFκB activation was more susceptible to inhibition by curcumin than DMC [22], suggesting that the phenyl methoxy groups contribute to antioxidant activity and the suppression of NFκB activation. These observations strongly indicate that DMC and curcumin suppressed nanoparticle-induced activation of NLRP3 inflammasome independent of their antioxidant and NFκB inhibitory activities.

The effects of DMC and curcumin are pleiotropic, and in line with this fact, a myriad of their potential molecular targets has been suggested including transcription factors, growth factors, protein kinases, and other enzymes [23,24]. Novelly, our molecular docking study proposed the NLRP3 NACHT domain as an additional molecular target for both compounds. The binding pocket was already defined in a previously reported 2.8 Å crystal structure of the NLRP3 NACHT domain in complex with an inhibitor, NP3-146 [14]. Therefore, the mechanism of action for DMC and curcumin can be extrapolated from the data for NP3-146. In addition, the proposed protein–ligand binding pose in our docking model can possibly aid in explaining the different efficacy profiles of DMC and curcumin (Figure 4). It is necessary to further corroborate the proposed docking models using in vitro experiments.

Although curcumin has been considered as a PAINS (pan-assay interference compounds), no clinical trial of curcumin has been successful [25]. This is due in part to its poor pharmacokinetic properties. However, recent advances in technology including hot-melt extrusion (HME) has significantly reduced this limitation by increasing bioavailability of curcumin [26]. In light of this fact, it would be of great significance to adopt a promising technology such as HME to enhance the solubility and bioavailability of the crude extract of *C. phaeocaulis*.

In conclusion, *C. phaeocaulis* was identified as an inhibitor of nanoparticle-activated NLRP3 inflammasome in macrophages and in an animal model of airway inflammation, which added another level to the in vitro and in vivo understanding of the anti-inflammatory effects of *C. phaeocaulis*. DMC and curcumin were found to contribute to the inflammasome-inhibitory activity of *C. phaeocaulis*. DMC exhibited slightly stronger potency than curcumin against nanoparticle-induced activation of NLRP3 inflammasome. This is in contrast to the previously reported higher antioxidant and NFκB inhibitory activities of curcumin. In the light of these results, both compounds seem to act independently of their antioxidant and NFκB inhibitory effects. Although a detailed structure–activity relationship study of curcumin is required, our in vitro and molecular docking studies showed that ortho-methoxy group on the aromatic ring of the compounds obviously affected biological activity of the compounds. These results indicate that the effects of DMC and curcumin are not just false positive results, which are often observed for PAINS.

## 4. Materials and Methods

### 4.1. Chemicals

Silicon dioxide (SiO_2_ nanopowder, 5–20 nm particle size), titanium dioxide (TiO_2_ nanopowder, 21 nm primary particle size), DMC (purity > 98% by HPLC), and curcumin (purity > 99.5% by HPLC) were purchased from Merck (Kenilworth, NJ, USA). MCC950 (purity > 98% by HPLC) was purchased from Cambridge Bioscience (Cambridge, UK).

### 4.2. Antibodies

Antibodies against IL-1β and cleaved IL-1β were purchased from Cell Signaling Technology (Danvers, MA, USA). Caspase-1 and cleaved caspase-1 (p20) were purchased from Abcam (Cambridge, UK).

### 4.3. Plant Material and Extract Preparation

The dried rhizomes of *Curcuma phaeocaulis* were purchased at a local market in Boeun, Korea in 2019 and its identity was authenticated by Jin Kyu Kim. A voucher specimen (Voucher No.: G77) was deposited in the herbarium at the Gyeonggido Business and Science Accelerator. The rhizomes (100 g) were pulverized and extracted with 70% ethanol (2 L) for 5 days, and then the extract was evaporated under reduced pressure.

### 4.4. Macrophage Differentiation and Inflammasome Activation

Human primary bone marrow mononuclear cells were purchased from ATCC, Manassas, VA, USA (catalog number PCS-800-013). The cells were plated onto either a 96-well plate (4 × 10^4^ cells/well) or a 12-well plate (5 × 10^5^ cells/well), and were differentiated to macrophages for seven days in RPMI1640 containing 10% FBS and 50 ng/mL M-CSF. The culture medium was then replaced with RPMI1640 containing 10% FBS and lipopolysaccharide (0.5 μg/mL). After 2 h of incubation, the cells were washed with RPMI-1640 and were pretreated with either DMSO, MCC950, or extracts for 1 h in RPMI-1640 without any supplement, and then were treated with either SiO_2_ (100 μg/mL) or TiO_2_ (100 μg/mL) nanoparticles for 3 h. The conditioned media were used for either ELISA using BD OptEIA Human IL-1β ELISA set (BD Bioscience, San Jose, CA, USA) or Western blot analysis.

### 4.5. Western Blot Analysis

The conditioned media derived from 12-well plates were concentrated approximately five times using Amicon centrifugal filter device (Millipore, Danvers, MA, USA; 3 kDa MWCO), and then used for Western blot analysis as previously described [27]. An equal amount of each sample was resolved on an SDS-polyacrylamide gel and electroblotted onto a PVDF membrane. The blots were blocked with 5% skim milk in PBS containing 0.05% Tween 20 and incubated with an appropriate antibody (diluted 1:1000 in blocking solution). Immobilon Western HRP Substrate kit (Merck, Kenilworth, NJ, USA) and ImageQuant LAS 4000 software (GE Healthcare, Chicago, IL, USA) were used to detect Chemiluminescence signals. Band intensities were quantified using ImageJ software. One representative Western blot for each experiment is shown in the figures. Data from three independent experiments were averaged for each condition and presented in the form of bar graphs. Data are expressed in fold changes compared with control (mean ± SD). Statistical analysis was performed using GraphPad Prism (GraphPad Software, San Diego, CA, USA), and *p*-values were calculated by paired *t*-test.

### 4.6. UHPLC-HR MS/MS Analysis of C. phaeocaulis Extract

*C. phaeocaulis* extract was separated on an ACQUITY UPLC BEH C_18_ column (2.1 × 150 mm, 1.7 μm; Waters Co., Wilmslow, UK). The mobile phases, A and B, were water and acetonitrile, respectively, containing 0.1% formic acid and the solvent gradient conditions were as follows: 5% B at 0–1 min, 5–70% B at 1–20 min, 70–100% B at 20–24 min, and 100% B at 24–27 min. Each compound was detected using a photodiode array at 200–500 nm. High-resolution mass spectra were acquired on an LTQ Orbitrap XL and analyzed with Xcalibur software (Thermo Fisher Scientific, Waltham, MA, USA).

### 4.7. Molecular Docking

The crystal structure of *Homo sapiens* ASC (PDB code: 2KN6) and NLRP3 NACHT domain (PDB code: 7ALV) co-crystallized with an inhibitor was chosen as templates for docking simulation, which was performed in Glide software (Maestro, version 12.9, Schrödinger, New York, NY, USA). LigPrep was used to generate 3D ligand structures of demethoxycurcumin (DMC) and curcumin. To characterize the putative binding sites, the prepared protein structures were submitted to the SiteMap module as implemented in Schrödinger Suite [28,29]. The active grid was generated using the receptor grid application in the Glide module. Docking was performed on a defined receptor grid using the standard precision mode of Glide [30,31]. Based on the results of individual docking runs for each molecule, results were considered consistent if at least three of the five docking runs produce similar ligand forms, among which the ligand forms with the best docking score were selected for further analyses.

### 4.8. Animal Care and Experimental Model for Lung Inflammation

Six-week-old female C57BL/6 mice were purchased from OrientBio (Seongnam, Korea) and maintained in specific pathogen-free conditions for 1 week for adaptation. SiO_2_ (20 mg/kg) and TiO_2_ (20 mg/kg) were administered by intratracheal instillation following anesthesia with pentobarbital (42 mg/kg, i.p.). For sham control, the mice were administered phosphate buffered saline by intratracheal instillation following anesthesia. Separate groups of mice (*n* = 5 in each group) were administered with either vehicle, MCC950, or *C. phaeocaulis* extract (p.o.). At 8 or 12 h after the nanoparticle instillation, the mice were euthanized and BAL fluid was collected following a tracheotomy. After centrifugation of the collected BAL fluid (1.2 mL) at 400× *g* for 10 min, the supernatants were kept at −80 °C until analysis of cytokine levels. The levels of IL-1β were analyzed by ELISA (Invitrogen, Carlsbad, CA, USA, catalog number 88-7013). The pellets were suspended in 0.2 mL of PBS, and aliquots of suspended cells were counted using a hemocytometer. The statistical significance was calculated with one-way ANOVA followed by Tukey’s multiple range test using Statistical Package for the Social Science, ver. 12.2 (SPSS Inc., Chicago, IL, USA). A *p* value less than 0.05 was considered statistically significant.

## Figures and Tables

**Table 1:** Identification of seven compounds from *C. phaeocaulis* extract.

RT	*m*/*z*	Formula	MS/MS	λmax	Compound
14.22	339.1222	C_20_H_19_O_5_	255, 245, 177, 147, 145	258, 426	Demethoxycurcumin
14.36	235.1688	C_15_H_23_O_2_	217, 189, 175, 161, 133	260, 297, 346	Curcumenol
14.48	369.1326	C_21_H_21_O_6_	285, 177, 161	261, 431	Curcumin
14.72	247.1325	C_15_H_19_O_3_	229, 123	300	Zederone
16.82	231.1374	C_15_H_19_O_2_	213, 203, 189, 173, 161, 83	266	Curzerenone
17.15	231.1374	C_15_H_19_O_2_	213, 203, 189, 173, 161, 83	259, 300	Furanodienone
19.23	219.1740	C_15_H_23_O	201, 191, 137	257, 328	Germacrone

## Data Availability

Data are contained within the article and Supplementary Materials.

## Supplementary Materials

The following supporting information can be downloaded at: https://www.mdpi.com/article/10.3390/molecules27072101/s1, Figure S1: The screening assay using a library of extracts derived from herbal medicinal plants, Figure S2: The effects of the identified seven compounds on SiO_2_-activated NLRP3 inflammasome, Figure S3: Molecular docking of demethoxycurcumin (DMC) and curcumin to ASC protein structure.

## Sample Availability

Samples of *C. phaeocaulis* extracts are available from the authors.
