# Supplementary material for: Curcuma phaeocaulis Inhibits NLRP3 Inflammasome in Macrophages and Ameliorates Nanoparticle-Induced Airway Inflammation in Mice
Source: Molecules. 2022 Mar 24;27(7):2101. doi: 10.3390/molecules27072101 (PMC9000720; doi:10.3390/molecules27072101)
Supplement: Supplementary file 1 [file molecules-27-02101-s001.zip › molecules-1630807-supplementary.pdf]

molecules

MDPI

Supplementary Material

Curcuma phaeocaulis Inhibits NLRP3 Inflammasome in Macrophages and Ameliorates Nanoparticle-Induced Airway Inflammation in Mice

Yeon-Ju Nam [1,†], Jiwon Choi [2,†], Jong Suk Lee [1,†], Changon Seo [1], Gyeongbeen Lee [1], Youngsu Lee [1], Jin Kyu Kim [1], Pansoo Kim [1], Jeong Ju Lim [3], Hyeon-Son Choi [4] and Yongmun Choi [1,*]

[1] Biocenter, Gyeonggido Business and Science Accelerator, Suwon 16229, Korea; nyj@gbsa.or.kr (Y.-J.N.); leejs@gbsa.or.kr (J.S.L.); changon2@gbsa.or.kr (C.S.); bin@gbsa.or.kr (G.L.); gju6020@naver.com (Y.L.); jinkyu90@gbsa.or.kr (J.K.K.); pan@gbsa.or.kr (P.K.)
[2] College of Pharmacy, Dongduk Women's University, Seoul 02748, Korea; edccjw@gmail.com
[3] Interdisciplinary Program in Precision Public Health, Korea University, Seoul 02841, Korea; hdgmp42jj@naver.com
[4] Department of Food Nutrition, Sangmyung University, Seoul 03016, Korea; hsc1970@smu.ac.kr
* Correspondence: ychoi@gbsa.or.kr; Tel.: +82-31-888-6972
† These authors contributed equally to this work.

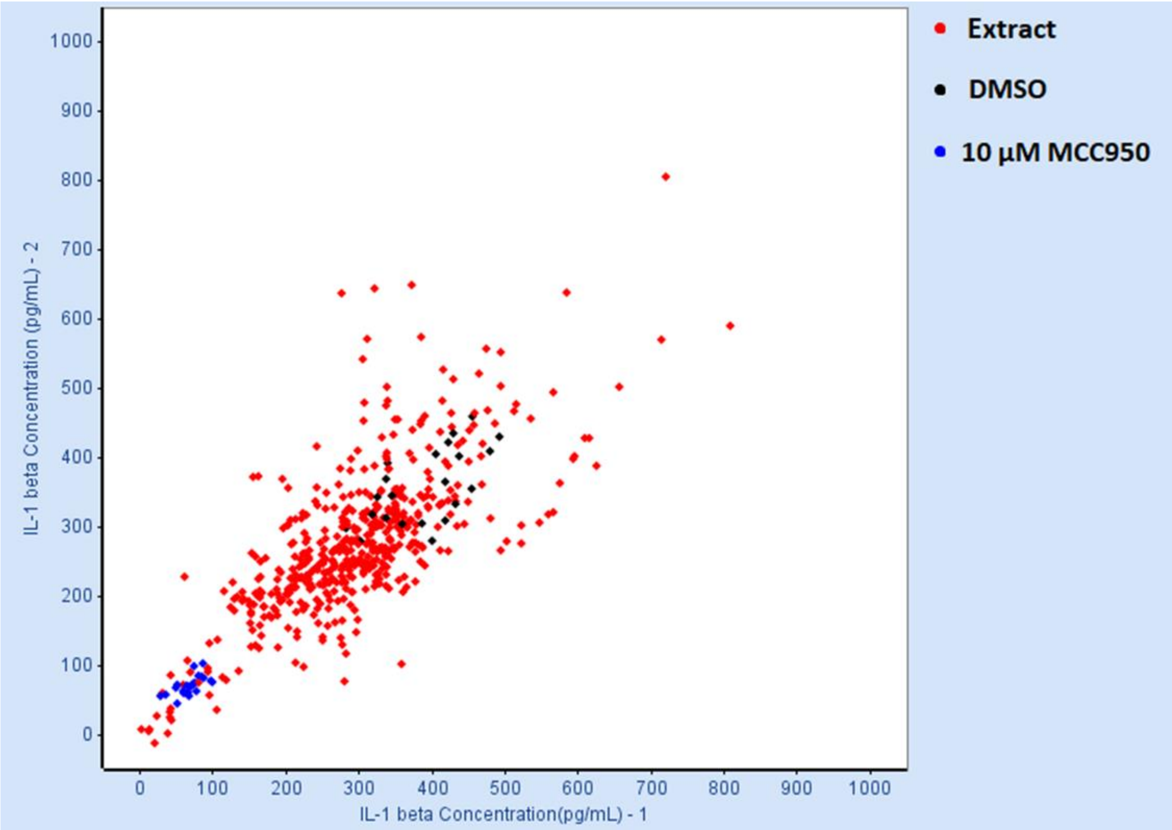

Figure S1. The screening assay using a library of extracts derived from herbal medicinal plants. Human bone marrow-derived mononuclear cells were differentiated into macrophages. The cells were primed with lipopolysaccharide and treated with SiO_2 to activate inflammasome. The effects of extracts on the activation of inflammasome was determined by analyzing the levels of the secreted IL-1β in cultured media by ELISA. Data from two independent replicates were plotted onto X (1st experiment) and Y (2nd experiment) axis on a scatter plot.

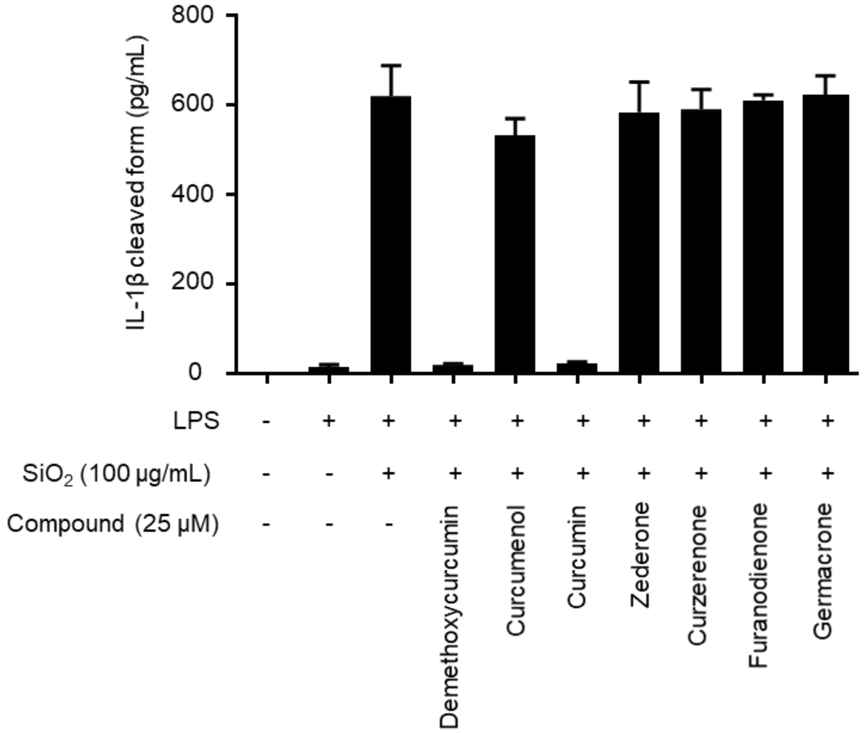

Figure S2. The effects of the identified seven compounds on SiO_2-activated NLRP3 inflammasome. Human bone marrow-derived mononuclear cells were differentiated into macrophages. The cells were primed with lipopolysaccharide and treated with SiO_2 to activate inflammasome. The effects of compounds on the activation of inflammasome was determined by analyzing the levels of the secreted IL-1β in cultured media by ELISA. Data from two independent replicates are shown as bar graphs. Data are expressed as mean ± standard deviation.

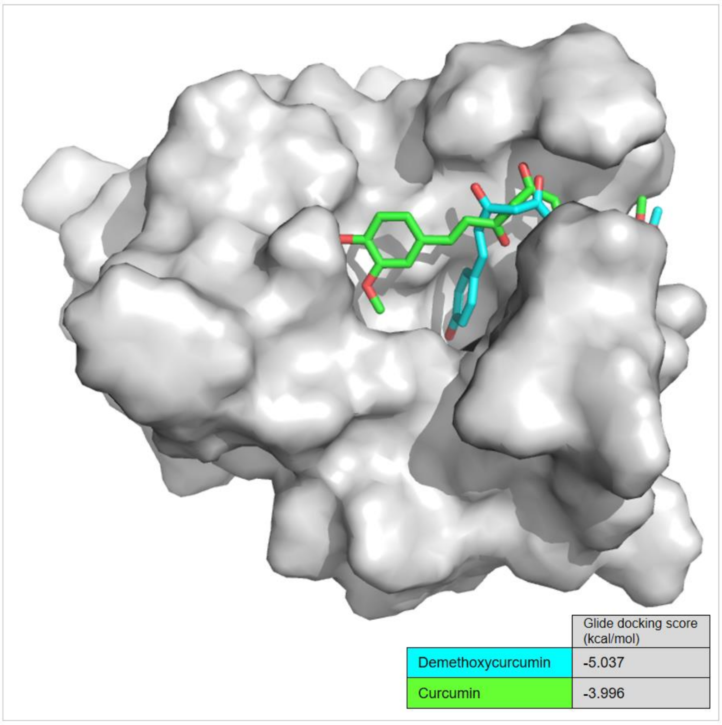

Figure S3. Molecular docking of demethoxycurcumin (DMC) and curcumin to ASC protein structure. Superimposition of predicted conformations for DMC (cyan) and curcumin (green) in ASC protein structure. Figures were drawn by using PyMol (Delano Scientific LLC, San Carlos, CA).
